# Waist-to-height ratio as a predictor of metabolic syndrome in a population with different degrees of glucose tolerance

**DOI:** 10.1186/1758-5996-7-S1-A149

**Published:** 2015-11-11

**Authors:** Tássia Cividanes Pazinato, Bárbara Limberger Nedel, Anize Delfino von Frankenberg, Vanessa Piccoli, Luciana Pavan Antoniolli, Mayara Abichequer Beer, Letícia Maria Tedesco Silva, Rodrigo Soares de Souza Marques, Leonardo de Andrade Mesquita, Monique de Moura Machado, André Fernandes Reis, Fernando Gerchman

**Affiliations:** 1 UFRGS, Porto Alegre, Brazil

## Background

Intra-abdominal fat (IAF) accumulation is related to metabolic syndrome (MS), type 2 diabetes mellitus (DM2) and cardiovascular disease (CVD). Among the indices that reflect IAF, waist-to-height ratio (WtHR) has been proposed as an index that can not only estimate IAF, but adjust it to body size, which possibly makes it an useful tool for risk prediction of MS and CVD.

## Objective

To compare the WtHR with other indices of central obesity and body fat distribution to identify MS.

## Materials and methods

We designed a cross-sectional study of consecutive individuals from 2 university hospitals of different Brazilian sites. Subjects (n=655, women 52.1%, 57.4±11.6 yrs.; mean±SD) were submitted to an evaluation that consisted of anthropometric assessment (BMI, WtHR, Waist-to-hip ratio and electric bioimpedanciometry), 2h 75g OGTT (estimation of insulin sensitivity index of Stumvoll [ISI]), lipids, A1c, fasting glucose, C-reactive protein (US-CRP), fibrinogen, adiponectin and ambulatory blood pressure measurement. MS was defined (MS 82.8%) according to the harmonization criteria performed by different medical societies. Patients were categorized by glucose tolerance status in normal glucose tolerance (NGT 24.1%), prediabetes (PDM 37.5%) and diabetes (DM 33.5%). A two-sided P value <0.05 was considered significant.

## Results

WtHR increased progressively with decreasing glucose tolerance status (NGT 0.59 vs. PDM 0.60 vs. DM 0.63; P<0.001) and with the presence of MS compared to the absence of MS (0.62 vs. 0.55; P<0.001). WtHR was positively related to US-CRP levels (r=0.521; P<0.001), total body fat (r=0.599; P<0.001), Stumvoll ISI (r=0.427; P<0.001), fibrinogen (r=0.275; P<0.001), triglycerides (r=0.239; P<0.001) and fasting glucose (r=0.109; P=0.006) and was inversely related to adiponectin levels (r=-0.143; P<0.001). ROC curve analyses showed that WtHR was superior to BMI (AUC 0.743 vs. 0.677; P<0.001), but similar to waist circumference (AUC 0.743 vs. 0.756; P=0.341) in predicting MS. The performance of WtHR was still greater than that of BMI after excluding waist circumference from MS criteria (AUC 0.639 vs. 0.576; P=0.005).

## Conclusion

WtHR was superior to BMI and similar to waist circumference in defining MS, suggesting that it may be used as a tool to discriminate subjects at greater risk of developing diabetes and cardiovascular disease.

